# Cryopreservation in 95% serum with 5% DMSO maintains colony formation and chondrogenic abilities in human synovial mesenchymal stem cells

**DOI:** 10.1186/s12891-019-2700-3

**Published:** 2019-07-06

**Authors:** Ryota Fujisawa, Mitsuru Mizuno, Hisako Katano, Koji Otabe, Nobutake Ozeki, Kunikazu Tsuji, Hideyuki Koga, Ichiro Sekiya

**Affiliations:** 1 Center for Stem Cell and Regenerative Medicine, Tokyo Medical and Dental University, 1-5-45 Yushima, Bunkyo-ku, Tokyo, 113-8510 Japan; 2 Department of Cartilage Regeneration, Tokyo Medical and Dental University, Tokyo, Japan; 3 Department of Joint Surgery and Sports Medicine, Tokyo Medical and Dental University, Tokyo, Japan

## Abstract

**Background:**

Synovial mesenchymal stem cells (MSCs) are an attractive cell source for cartilage and meniscus regeneration. The optimum cryopreservation medium has not been determined, but dimethylsulfoxide (DMSO) should be excluded, if possible, because of its toxicity. The purposes of our study were to examine the possible benefits of higher concentrations of serum and the effectiveness of 100% serum (without DMSO) for the cryopreservation of synovial MSCs.

**Methods:**

Human synovium was harvested from the knees of four donors with osteoarthritis during total knee arthroplasty. Synovial MSCs (8 × 10^5^ cells) were suspended in 400 μL medium and used as a Time 0 control. The same number of synovial MSCs was also suspended in 400 μL α-MEM medium containing 10% fetal bovine serum (FBS) (5% DMSO, and 1% antibiotic), 95% FBS (and 5% DMSO), or 100% FBS (no DMSO) and cryopreserved at − 80 °C for 7 days. After thawing, the cell suspensions (1.5 μL; 3 × 10^3^ cells) were cultured in 60 cm^2^ dishes for 14 days for colony formation assays. Additional 62.5 μL samples of cell suspensions (1.25 × 10^5^ cells) were added to tubes and cultured for 21 days for chondrogenesis assays.

**Results:**

Colony numbers were significantly higher in the Time 0 and 95% FBS groups than in the 10% FBS group (*n* = 24). Colony numbers were much lower in the 100% FBS group than in the other three groups. The cell numbers per dish reflected the colony numbers. Cartilage pellet weights were significantly heavier in the 95% FBS group than in the 10% FBS group, whereas no difference was observed between the Time 0 and the 95% FBS groups (*n* = 24). No cartilage pellets formed at all in the 100% FBS group.

**Conclusion:**

Synovial MSCs cryopreserved in 95% FBS with 5% DMSO maintained their colony formation and chondrogenic abilities to the same levels as observed in the cells before cryopreservation. Synovial MSCs cryopreserved in 100% FBS lost their colony formation and chondrogenic abilities.

**Electronic supplementary material:**

The online version of this article (10.1186/s12891-019-2700-3) contains supplementary material, which is available to authorized users.

## Background

Human synovial mesenchymal stem cells (MSCs) are a promising cell source for regeneration of cartilage and meniscus [1]. In clinical situations, transplantation of synovial MSCs can regenerate cartilage defects in the knee [2]. For this clinical study, we used synovial MSCs just after harvesting. If synovial MSCs could be cryopreserved in a condition that maintained their activity, this would greatly facilitate these transplantations.

The cryopreservation medium utilized thus far for synovial MSC culture used in both basic [3, 4] and translational research [5, 6] has typically been a basic culture medium, such as Dulbecco’s modified Eagle medium (DMEM) or α-minimum essential medium (α-MEM), with added fetal bovine serum (FBS) and dimethyl sulfoxide (DMSO). However, an optimum cryopreservation medium has yet to be identified. We recently reported that synovial MSCs maintained their viability as well as their chondrogenic ability for 48 h when preserved in 100% human serum at 4 °C or 13 °C [7]. This suggests that higher concentrations of serum in the cryopreservation medium improves cell viability.

Our first purpose in the present study was to examine whether a higher concentration of serum in the cryopreservation medium would be beneficial for the preservation of human synovial MSCs. We also focused on DMSO, as it is widely used as a cryoprotective agent; however, it has toxic effects [8–11] and the safety of intraarticular transplantation of DMSO-containing material is not established. If DMSO could be excluded from the cryopreservation medium, then synovial MSCs could be transplanted into the knee joint directly after thawing without requiring any procedure for DMSO removal. Our second purpose was therefore to examine whether 100% serum (without any DMSO) might serve as an effective cryopreservation medium for synovial MSCs.

## Methods

### Synovial MSCs

This study was approved by the Medical Research Ethics Committee of Tokyo Medical and Dental University, and informed consent was obtained from all study subjects. Human synovium was harvested from the knees of four female donors with osteoarthritis (OA) during total knee arthroplasty operations. The average age and standard deviation of the patients was 71 ± 6 years. The synovium was minced and digested in a solution of 3 mg/mL collagenase (Sigma-Aldrich Japan, Tokyo, Japan) at 37 °C for 3 h and the digested cells were filtered through a 70 μm cell strainer (Greiner Bio-One GmbH, Frickenhausen, Germany). The obtained nucleated cells were suspended in α-MEM (Thermo Fisher Scientific, Rockford IL, USA) supplemented with 1% antibiotic-antimycotic (Thermo Fisher Scientific) and 10% FBS (Thermo Fisher Scientific) and cultured in a cell culture incubator (Astec Co. Ltd., Fukuoka, Japan) in 5% CO_2_ at 37 °C for 14 days. When the MSCs reached 70–80% confluence, they were replated continuously. Synovial MSCs of passage 2 were used in this study.

### Flow cytometry

For cultured synovial MSCs from four donors, surface markers were examined using a FACS Verse instrument (Becton, Dickinson and Company; BD, NJ, USA). The cells before cryopreservation were suspended in FACS buffer using Hank’s balanced salt solution (HBSS) at a density of 5 × 10^5^ cells/mL and stained for 30 min with the antibodies CD44 (PE-Cy7), CD45 (APC-H7), CD73 (V450), CD90 (PE), CD105 (APC) (all from BD), and Ghost Dye Violet 510 for dead cells (Tonbo Biosciences, CA, USA). FlowJo software (Tree Star Software, CA, USA) was used for the analysis.

### Differentiation assays

For chondrogenesis, 1.25 × 10^5^ synovial MSCs were suspended in 0.5 mL chondrogenic induction medium consisting of DMEM (Thermo Fisher Scientific) supplemented with 10 ng/mL transforming growth factor-β3 (TGF-β3, Miltenyi Biotec, Bergisch Gladbach, Germany), 500 ng/mL bone morphogenetic protein 2 (BMP-2, Medtronic, Minneapolis, MN, USA), 40 μg/mL proline, 100 nM dexamethasone, 100 μg/mL pyruvate, 50 ng/mL ascorbate-2-phosphate, and 50 mg/mL ITS Premix (Becton Dickinson, San Jose, CA, USA). The cells were pelleted by centrifugation at 450×g for 10 min and then cultured for 21 days. After 21 days, the pellets were sectioned and stained with toluidine blue (Fujifilm Wako Pure Chemical Corporation, Osaka, Japan).

For adipogenesis, 100 synovial MSCs were plated in a 60 cm^2^ dish and cultured for 14 days in culture medium to make cell colonies. The adherent cells were further cultured in adipogenic induction medium consisting of α-MEM supplemented with 100 nM dexamethasone, 0.5 mM isobutyl-methylxanthine (Sigma-Aldrich), and 50 mM indomethacin (Fujifilm, Wako Pure Chemical Corporation) for an additional 21 days. Adipocytes were stained with oil red O (Muto Pure Chemicals, Tokyo, Japan).

For calcification, 100 synovial MSCs were plated in a 60 cm^2^ dish and cultured for 14 days in culture medium to make cell colonies. The adherent cells were further cultured in calcification induction medium consisting of α-MEM supplemented with 50 μg/mL ascorbic acid 2-phosphate (Fujifilm Wako Pure Chemical Corporation), 10 nM dexamethasone (Fujifilm Wako Pure Chemical Corporation), and 10 mM β-glycerophosphate (Sigma-Aldrich). After 21 days, calcification was assessed by alizarin red staining (Merck Millipore, Billerica, MA, USA).

### Freezing and thawing methods

**Fig. 1:**
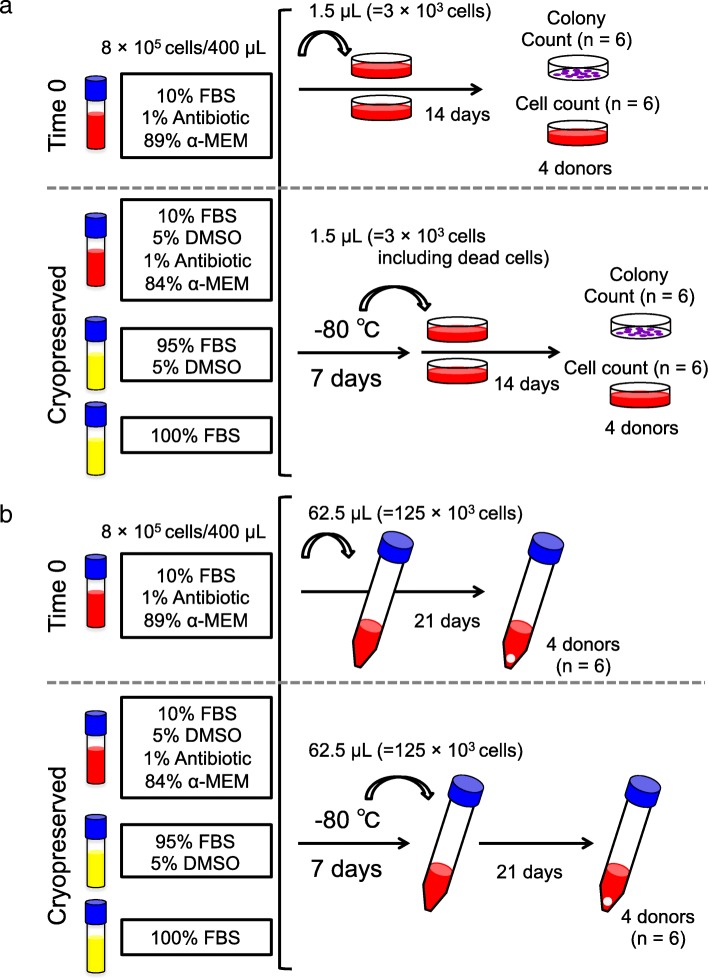
Scheme for the colony formation and chondrogenesis assays. **a** Colony formation assay. Synovial mesenchymal stem cells (MSCs; 8 × 10^5^ cells) were suspended in 400 μL medium containing 10% fetal bovine serum (FBS) as a time 0 control. Synovial MSCs (8 × 10^5^ cells) suspended in 400 μL medium containing 10% FBS, 95% FBS, or 100% FBS were cryopreserved at − 80 °C for 7 days and thawed. A 1.5 μL volume of cell suspension (containing 3 × 10^3^ cells, including living and dead cells) was added to 60 cm^2^ dishes for colony formation and cell proliferation assays. **b** Chondrogenesis assay. A 62.5 μL volume of cell suspension (containing 1.25 × 10^5^ cells, including living cells and dead cells) was added to 15 mm tubes for chondrogenesis assays

8 × 10^5^ synovial MSCs were allocated into 4 freezing tubes (Sumitomo Bakelite, Tokyo, Japan) and suspended in 400 μL medium (Fig. 1). For the “Time 0” group, the cells were suspended in medium consisting of 10% FBS, 1% Antibiotic, and 89% α-MEM. For the “10% FBS” group, the cells were suspended in medium consisting of 10% FBS, 5% DMSO (CultureSure DMSO; Fujifilm Wako Pure Chemical Corporation), 1% Antibiotic, and 84% α-MEM. For the “95% FBS” group, the cells were suspended in medium consisting of 95% FBS and 5% DMSO. For the “100% FBS” group, the cells were suspended in 100% FBS. The tubes in the “10% FBS” group, the “95% FBS” group, and the “100% FBS” group were put in a bio freezing vessel (Bicell, Japan Freezer, Tokyo, Japan), and kept in a freezer at − 80 °C for 7 days (Fig. 1). Then, the tubes were removed out of the freezing vessel and the frozen cells in the tube were thawed using a frozen cell thawing device (ThawSTAR, Astero Bio, Menlo Park CA, USA).

### Colony formation

A 1.5 μL volume of cell suspension (containing 3 × 10^3^ cells, including living cells and dead cells) was added to twelve 60 cm^2^ dishes, 10 mL α-MEM containing 10% FBS was added to each dish, and the cells were cultured for 14 days (Fig. 1a). Six dishes were stained with crystal violet to count the total numbers of cell colonies. Only colonies greater than 2 mm in diameter or showing distinct staining were counted. Cells were harvested from the other 6 dishes, and the cell numbers per dish were counted with a hemocytometer. The cell number per colony was determined by counting the cell number per dish for 6 dishes (dishes A, B, C, D, E, and F), and the colony numbers per dish were counted for 6 additional dishes (dishes G, H, I, J, K, and L). The cell number per colony was calculated based on the cell number from dish A divided by the colony number from dish G, and this calculation was repeated for the remaining pairs of dishes (i.e., B and H, C and I, D and J, E and K, and F and L.) The mean and standard deviation were then determined for cell number per colony (4 donors, *n* = 24) [12].

### Comparison of chondrogenesis potential

Synovial MSCs (8 × 10^5^ cells) were suspended in 400 μL medium containing 10% FBS, 95% FBS, or 100% FBS, cryopreserved at − 80 °C for 7 days, and then thawed. A 62.5 μL volume of cell suspension (containing 1.25 × 10^5^ cells, including living and dead cells) was added to six 15 mL tubes (Falcon) containing 0.5 mL chondrogenic induction medium. The cells were pelleted by centrifugation at 450 × g for 10 min and cultured for 21 days (Fig. 1b). The cultured cell pellets were photographed and weighed with a semi micro balance (CPA225D, Sartorius, Gottingen, Germany). The pellets were sectioned and stained with toluidine blue and immunostained with collagen type II (Kyowa Pharma Chemical Co., LTD, Toyama, Japan). The mean and standard deviation were then determined for the pellet weight (4 donors, *n* = 24).

### Statistical analysis

All data were statistically evaluated with the Friedman test and Dunn’s multiple comparison test using GraphPad Prism 6 (GraphPad Software, CA, USA). Data were expressed as mean ± standard deviation (SD). Two-tailed *p* values < .05 were considered statistically significant.

## Results

### MSC characteristics

**Fig. 2:**
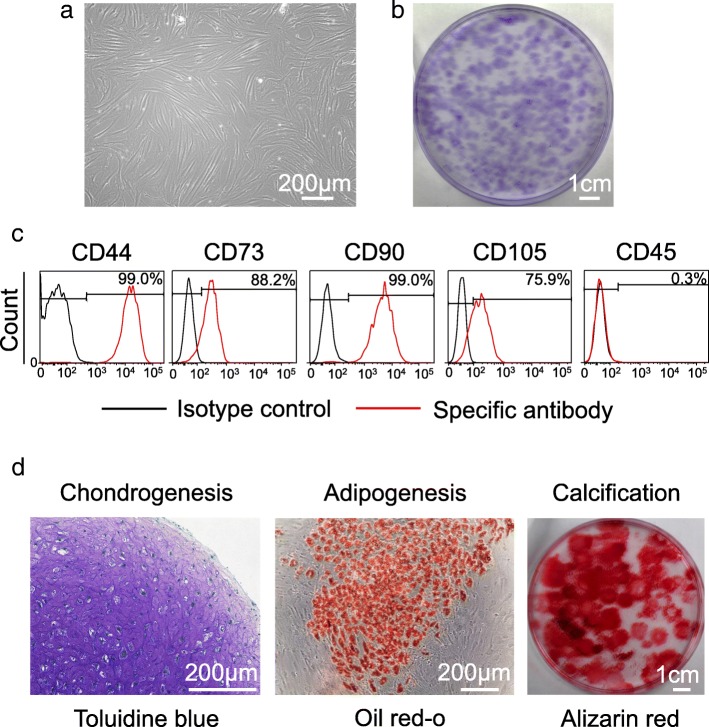
Characteristics of synovial mesenchymal stem cells (MSCs) as MSCs. **a** Cell morphology. **b** Colony morphology. **c** Representative histograms for surface markers (d) Multidifferentiation

Synovial cells were spindle shaped (Fig. 2a) and formed cell colonies 14 days after the initial plating (Fig. 2b). They stained positive for CD 44, 73, 90, and 105 and negative for CD 45 (Fig. 2c). They showed chondrogenesis, adipogenesis, and calcification potential (Fig. 2d). Overall, they had characteristics of MSCs [13].

### Colony formation

**Fig. 3:**
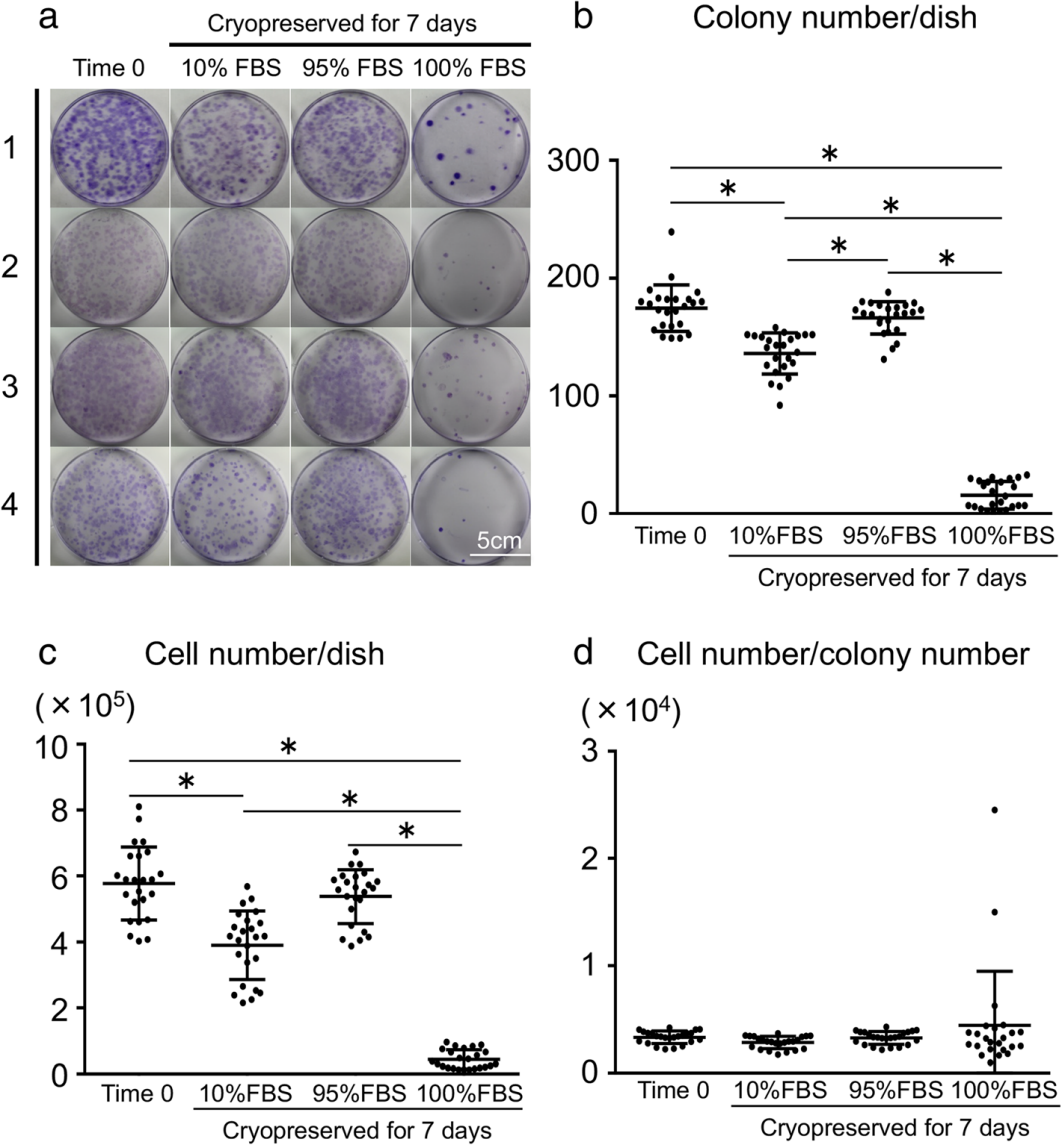
Analysis of colony formation. **a** Representative dishes stained with crystal violet. Synovial mesenchymal stem cells (MSCs) were derived from four donors. **b** Colony numbers per dish. Data are shown as means ± SD (*n* = 4 for each donor). *p < .05 by the Friedman test followed by Dunn’s multiple comparisons. **c** Cell numbers per dish. **d** Cell numbers per colony

Colony formation was poor in the 100% FBS group (Fig. 3a). The colony numbers per dish were significantly higher in the Time 0 group and in the 95% FBS group than in the 10% FBS group (Fig. 3b). The colony numbers per dish were much lower in the 100% FBS group than in the other three groups. Similar differences were obtained for cell numbers per dish (Fig. 3c). No statistically significant differences were noted for cell numbers per colony among the four groups (Fig. 3d). Each donor analysis yielded similar results (Additional file 1: Figure S1).

### Chondrogenesis

**Fig. 4:**
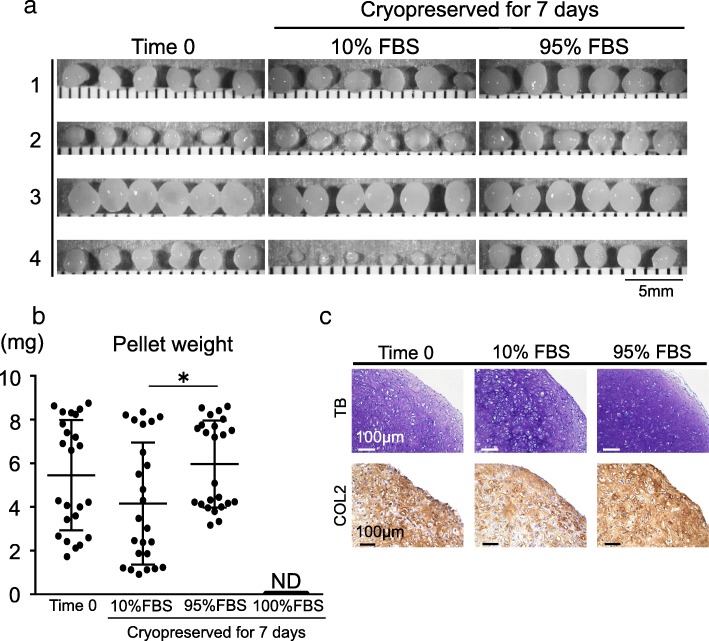
Analysis of chondrogenesis. **a** Representative macroscopic appearance of cartilage pellet. Synovial mesenchymal stem cells (MSCs) were derived from four donors. In the 100% fetal bovine serum (FBS) group, no cartilage pellets were formed. **b** Pellet weight. Data are shown as means ± SD (n = 4 for each donor). *p < .05 by the Friedman test followed by Dunn’s multiple comparisons. ND: not detected. **c** Representative histological sections stained with toluidine blue and immunostained for collagen type II

Cartilage pellets were obtained (Fig. 4a) for all except the 100% FBS group. The pellet weight was significantly heavier in the 95% FBS group than in the 10% FBS group, but no significant difference was noted between the Time 0 group and the 95% FBS group (Fig. 4b). The obtained cartilage pellets showed positive staining with toluidine blue and collagen type II (Fig. 4c). For each donor analysis, almost identical results were obtained, with no statistically significant difference (Additional file 2: Figure S2).

## Discussion

We examined the effect of the cryopreservation medium composition on the maintenance of the colony formation and chondrogenic abilities of synovial MSCs. Cryopreservation of human synovial MSCs in 95% FBS with 5% DMSO maintained these abilities at the same level as that observed in the cells before cryopreservation. Preservation of human synovial MSCs in 100% FBS (without any DMSO) resulted in extensive loss of colony formation ability and a complete loss of chondrogenic ability.

The most common cellular damage caused by freezing occurs because of the formation of ice crystals, which form around 0 °C and destroy cell membranes [14]. Here, a higher concentration of serum in the cryopreservation medium resulted in a better preservation of the synovial MSCs. This is possibly due to a decrease in moisture in the cells in response to adjustments in osmotic pressure [15], as well as to a reduction in the occurrence of ice crystals due to the added FBS. More than half of the serum protein is albumin, which can buffer the pH of the solution and maintain the osmotic pressure [16], and thereby function as a cryoprotectant.

Another frequently used cryoprotectant is DMSO, but its use in mammals is limited because of its toxicity. In four species (mice, rats, cats, dogs), the LD50s are between 2.5 and 8.9 g/kg for DMSO administered intravenously. The symptoms at near lethal doses are similar and include spontaneous motor activity, tremors, myasthenia, prostration, transient convulsion, dyspnea, pulmonary edema, and hemorrhaging [17]. Yellowlees et al. reported a toxic reaction in two elderly people receiving an intravenous dosage of 100 g of 20% DMSO for three days for the treatment of arthritis. One patient experienced serious illness, including oliguria, hemolysis, tremor, and loss of consciousness, whereas the second patient did not become ill [18]. O’Donnell et al. reported a patient with myeloblastic leukemia received intravenous cryopreserved autologous marrow blood containing 35 g of DMSO as a 10% solution and suffered a reaction to the administration. In addition to a drop in hemoglobin, the patient became agitated, pyretic, hypotensive, and tachycardic and died ten days later [19]. High doses of DMSO are therefore potentially dangerous. In our case, 8 × 10^5^ cells were suspended in 400 μL cryopreservation medium containing 5% DMSO. If 2.4 × 10^7^ cells were to be administered, the 12 mL of cryopreservation medium would corresponds 66 mg of DMSO, as the specific gravity of DMSO is 1.1. This would be a safe amount for intravenous administration, but whether it would be safe for intra-articular administration is unknown.

Cryoprotectants are classified as either permeating (DMSO is this type) or non-permeating, depending on their ability to traverse the cell membrane [20]. Non-permeating cryoprotectants include polyvinylpyrrolidone [21], sugars such as trehalose [22], sucrose [23], lactose [24], glucose [25], sugar alcohols (such as mannitol [26] and sorbitol [27]), and the polymer hydroxyethyl starch (HES) [28]. In the future, our intention is to compare the optimal conditions we determined in the present study with those obtained using these non-permeating cryoprotectants.

In the present study, the culture medium for the 10% FBS group contained 1% antibiotics, whereas the media for the 95 and 100% FBS groups did not. We initially hypothesized that 100% FBS would function as a cryopreservation medium because 100% FBS worked as a preservation medium [7]. We also wanted to examine the additional effect of DMSO (i.e., in the 95% FBS group). We also desired to compare the 100% FBS group to the 5% FBS group as the latter was cultured in our standard cryopreservation medium (containing 1% antibiotics, 5% DMSO, and 84% α-MEM). Contrary to our hypothesis, cryopreservation in 100% FBS was not successful. Some cell culture studies have reported that antibiotics affected cellular DNA synthesis as well as protein synthesis [29], while others found that antibiotics had minimal detrimental effects on cultured cells [30]. Some microorganisms can withstand low temperatures, so the risk of bacterial contamination cannot be denied even during cryopreservation [31]. For cell transplantation in clinical situations, antibiotics may be needed in the cryopreservation medium for safety. We will investigate the effect of antibiotics in cryopreservation media with high serum concentrations in future work.

Our colony formation and chondrogenesis assays were conducted by adding cell suspensions directly to dishes or tubes, without first determining the numbers of viable cells, as we considered this a more accurate method for evaluating cell viability. Our previous studies indicated that the statistical dispersions of the live cell rate, as determined by live/dead assays, and the apoptosis rate, as determined by flow cytometry, were relatively wide for human synovial MSCs after 48 h of preservation in Ringer’s solution or in complete human serum at 4, 13, and 37 °C [7].

The condition of cryopreservation did not affect the cell numbers per colony, though it did affect the colony numbers per dish and the cell numbers per dish. This indicates the possibility that a fraction of the cells was resistant to poor culture conditions, and these cells had a comparable colony formation ability. However, the actual number of these cells was quite low.

The chondrogenesis potential of MSCs was evaluated based on the pellet weight. Our findings indicated that the pellet increased in diameter, weight, and amount of cartilage matrix synthesized during the in vitro chondrogenesis of MSCs. Contrary, the DNA yield per pellet decreased. The amount of DNA per cell, as assessed by pre-labeling with 3H-thymidine, was maintained during the in vitro chondrogenesis of MSCs [32]. Our results indicate that the increase in pellet weight can be caused by the production of extracellular matrix, rather than the proliferation of the cells. We also previously reported that pellet weight was correlated with the expression of chondrogenic-specific genes and the amounts of glycosaminoglycans [33–35]. We believe that the pellet weight is therefore a convincing indicator of in vitro chondrogenesis in a population of MSCs [1, 7, 36].

Our study had three main limitations. We cryopreserved cells in a freezer at − 80 °C, rather than in a liquid nitrogen tank at − 150 °C, and the period of cryopreservation was only 7 days. Had we conducted the cryopreservation in a liquid nitrogen tank for longer durations, our outcomes might have been different. Kim et al. reported that the viability of human umbilical cord MSCs cryopreserved in a liquid nitrogen tank for 1 year was comparable to that of cells cryopreserved for 7 days [37]. Abbruzzese at al. reported that hematopoietic stem cells cryopreserved in a medium containing 5% DMSO in a liquid nitrogen tank remained viable for 10 years [38]. Since cryopreservation seems to be less effective at − 80 °C than − 150 °C, this suggests that our cryopreservation medium containing 95% FBS and 5% DMSO might allow storage of synovial MSCs for quite long periods at − 150 °C.

A second limitation was that we examined the effect of DMSO only at a 5% concentration. Thirumala et al. reported that DMSO concentrations ranging between 2 and 10% had the same effect on the viability of cryopreserved hepatocytes, whereas DMSO at less than 2% decreased viability [39]. This suggests that we could possibly reduce the concentration of DMSO for cryopreservation of synovial MSCs. In forthcoming studies, we will examine variations in DMSO concentrations to determine the minimum amount of DMSO required for cryopreservation of synovial MSCs.

A third limitation was our use of FBS instead of autologous human serum, which we used in our clinical study [2]. In comparison with FBS, autologous human serum is safer in clinical cell transplantation because it can prevent immune reactions and contamination with pathogens, such as prions or zoonotic viruses [40–42]. Here we did not use autologous human serum because it requires additional work. Since human serum has shown effects comparable to FBS in terms of the proliferation, chondrogenesis [43], and preservation [7] of synovial MSCs, we expect that human serum would have the same effect as FBS with respect to cryopreservation.

## Conclusions

Synovial MSCs cryopreserved in 95% FBS with 5% DMSO maintained their colony formation and chondrogenic abilities at the same level as that observed in the cells before cryopreservation. Synovial MSCs cryopreserved in 100% FBS (without any DMSO) lost their colony formation and chondrogenic abilities.

## Additional files

Additional file 1: **Figure S1.** Donor specific analysis of colony formation. (a) Colony number/dish. (b) Cell number/dish. (c) Cell number/colony number. (ZIP 507 kb)

Additional file 2: **Figure S2.** Patient-specific analysis of chondrogenesis. (PDF 268 kb)

## Data Availability

All the data supporting our findings are contained within the manuscript.
