# Supplementary figures and images for: Cryopreservation in 95% serum with 5% DMSO maintains colony formation and chondrogenic abilities in human synovial mesenchymal stem cells
Source: BMC Musculoskelet Disord. 2019 Jul 6;20:316. doi: 10.1186/s12891-019-2700-3 (PMC6612159; doi:10.1186/s12891-019-2700-3)

Supplementary Figure 1 (a)

Colony number/dish

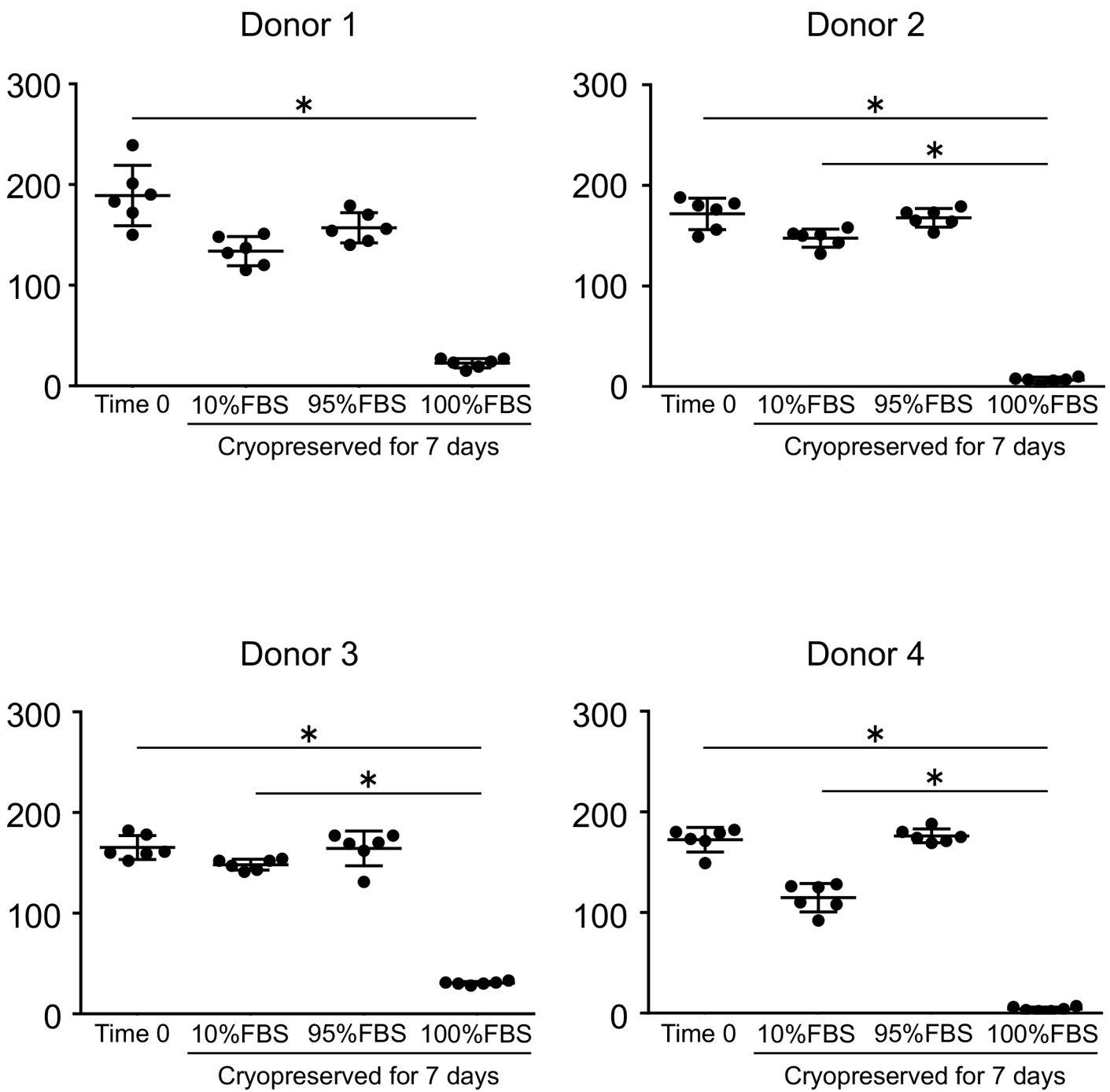

Supplement: Supplementary file 1 — Figure S1. Donor specific analysis of colony formation. (a) Colony number/dish. (b) Cell number/dish. (c) Cell number/colony number. (ZIP 507 kb) [file 12891_2019_2700_MOESM1_ESM.zip › S1aR4.pdf]

Supplementary Figure 1 (b)

Cell number/dish

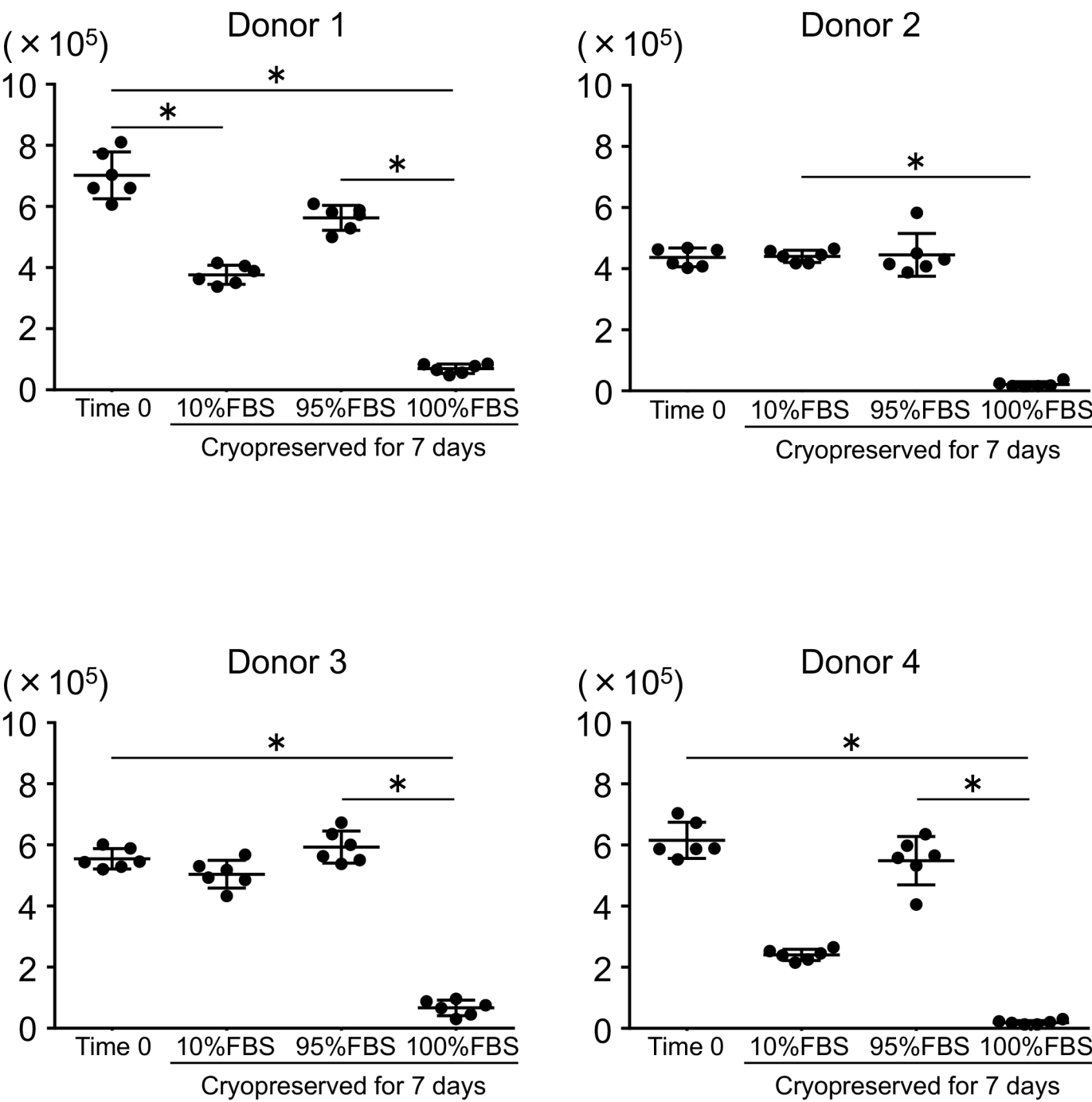

Supplement: Supplementary file 1 — Figure S1. Donor specific analysis of colony formation. (a) Colony number/dish. (b) Cell number/dish. (c) Cell number/colony number. (ZIP 507 kb) [file 12891_2019_2700_MOESM1_ESM.zip › S1bR4.pdf]

Supplementary Figure 1 (c)

Cell number/colony number

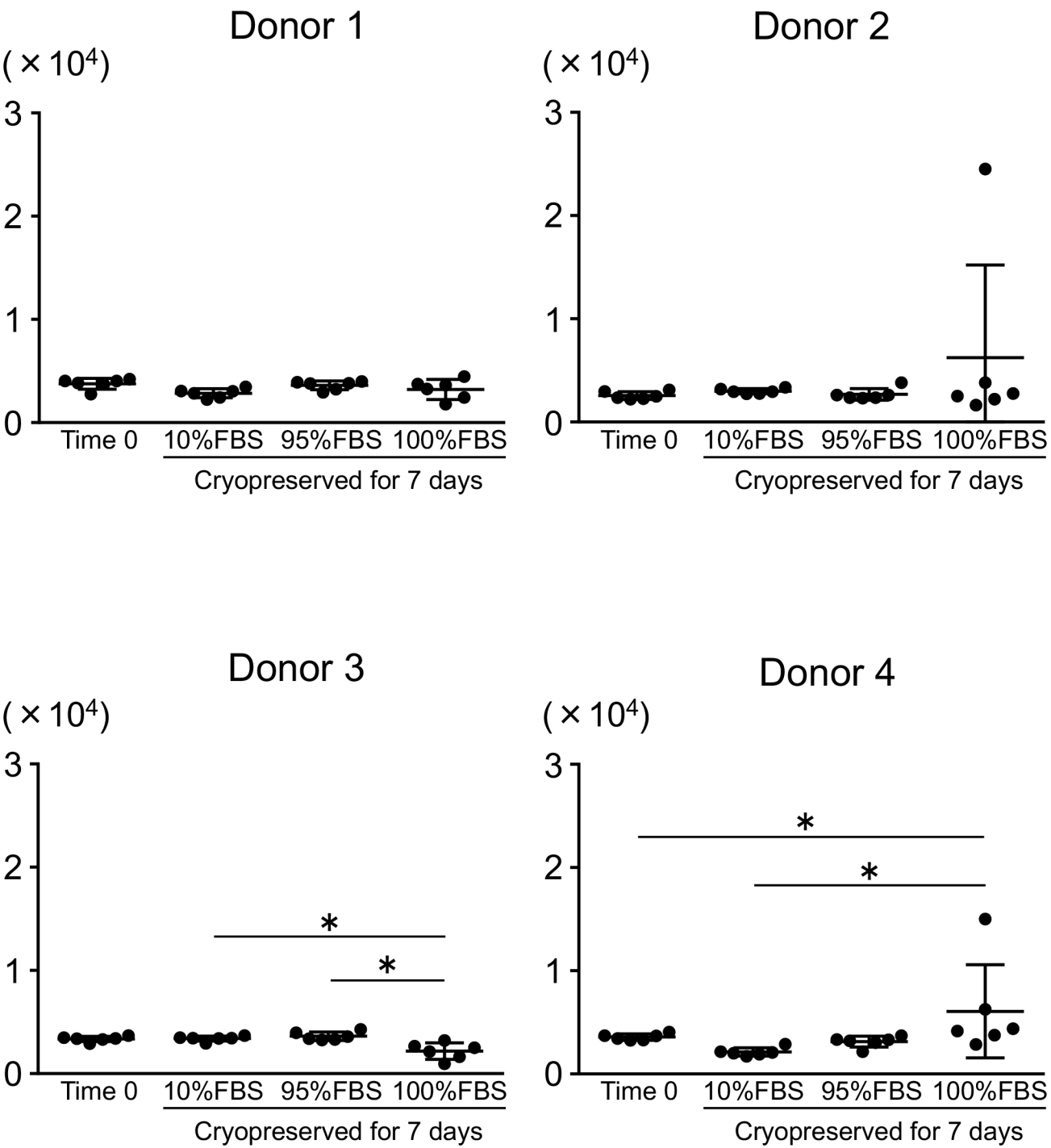

Supplement: Supplementary file 1 — Figure S1. Donor specific analysis of colony formation. (a) Colony number/dish. (b) Cell number/dish. (c) Cell number/colony number. (ZIP 507 kb) [file 12891_2019_2700_MOESM1_ESM.zip › S1cR4.pdf]

# Supplementary Figure 2

## Pellet weight

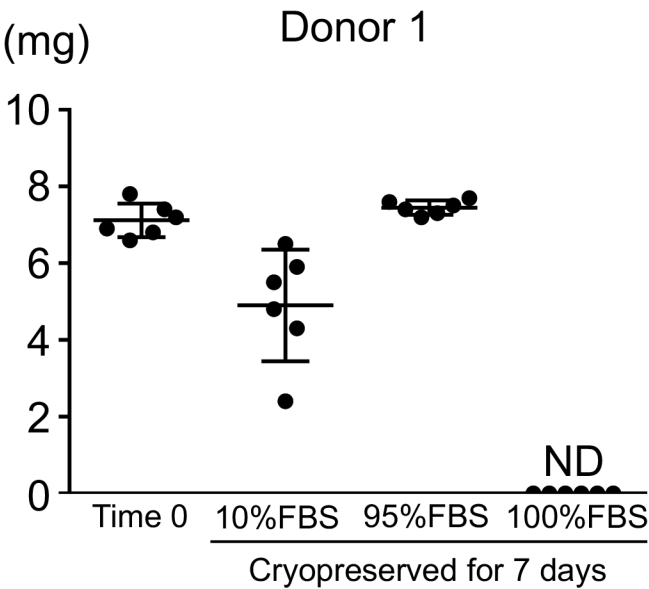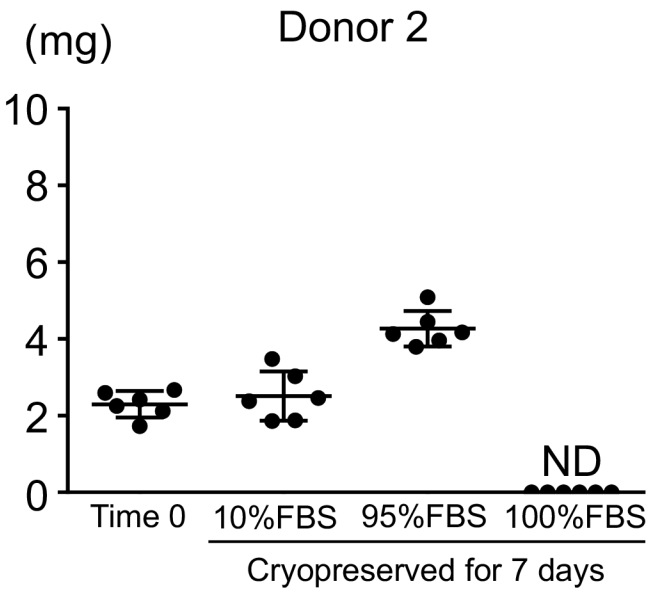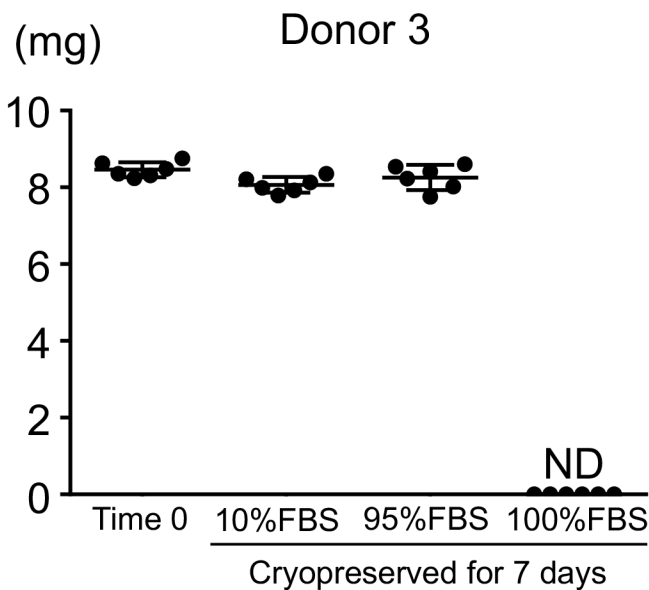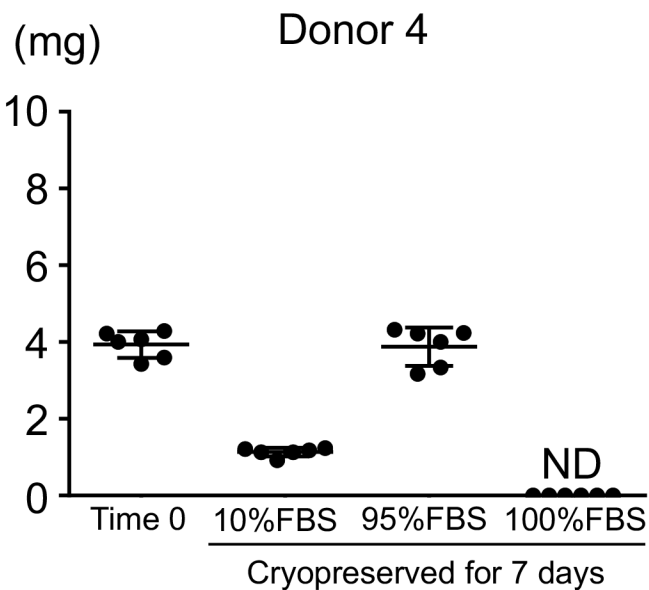

Supplement: Supplementary file 2 — Figure S2. Patient-specific analysis of chondrogenesis. (PDF 268 kb) [file 12891_2019_2700_MOESM2_ESM.pdf]
